# The prognostic value of URR equals that of Kt/V for all-cause mortality in Taiwan after 10-year follow-up

**DOI:** 10.1038/s41598-023-35353-8

**Published:** 2023-06-01

**Authors:** Yi-Kong Chen, Chih-Sheng Chu, Sheng-Wen Niu, Hugo You-Hsien Lin, Pei-Hua Yu, Feng-Ching Shen, Yu-Lin Chao, I-Ching Kuo, Chi-Chih Hung, Jer-Ming Chang

**Affiliations:** 1 Division of Nephrology, Department of Internal Medicine, Kaohsiung Medical University Hospital, Kaohsiung Medical University, Kaohsiung, Taiwan; 2 Department of Internal Medicine, Kaohsiung Medical University Hospital, Kaohsiung Medical University, Kaohsiung, Taiwan; 3 Division of Cardiology, Department of Internal Medicine, Kaohsiung Municipal Ta-Tung Hospital, Kaohsiung, Taiwan; 4 Division of Cardiology, Department of Internal Medicine, Kaohsiung Medical University Hospital, Kaohsiung Medical University, Kaohsiung, Taiwan; 5 Department of Internal Medicine, Kaohsiung Municipal Ta-Tung Hospital, Kaohsiung, Taiwan; 6 Faculty of Medicine, College of Medicine, Kaohsiung Medical University, Kaohsiung, Taiwan

**Keywords:** Kidney, Kidney diseases, Renal replacement therapy

## Abstract

Kt/V and URR (urea reduction ratio) measurements represent dialysis adequacy. Single-pool Kt/V is theoretically a superior method and is recommended by the Kidney Disease Outcomes Quality Initiative guidelines. However, the prognostic value of URR compared with Kt/V for all-cause mortality is unknown. The effect modifiers and cut-off values of the two parameters have not been compared. We investigated 2615 incident hemodialysis patients with URR of 72% and Kt/V (Daugirdas) of 1.6. The average patient age was 59 years, 50.7% were female, and 1113 (40.2%) died within 10 years. URR and Kt/V were both positively associated with nutrition factors and female sex and negatively associated with body weight and heart failure. In Cox regression mod-els for all-cause mortality, the hazard ratios (HRs) of high URR groups (65–70%, 70–75%, and > 75%) and the URR < 65% group were 0.748 (0.623–0.898), 0.693 (0.578–0.829), and 0.640 (0.519–0.788), respectively. The HRs of high Kt/V groups (Kt/V 1.2–1.4, 1.4–1.7, and > 1.7) and the Kt/V < 1.2 group were 0.711 (0.580–0.873), 0.656 (0.540–0.799), and 0.623 (0.498–0.779), respec-tively. In subgroup analysis, Kt/V was not associated with all-cause mortality in women. The prognostic value of URR for all-cause mortality is as great as that of Kt/V. URR > 70% and Kt/V > 1.4 were associated with a higher survival rate. Kt/V may have weaker prognostic value for women.

## Introduction

Dialysis inadequacy affects the morbidity and mortality of hemodialysis (HD) patients^1,2^. Dialysis inadequacy can be evaluated in multiple manners, including clearance of small and middle molecules^3,4^, acid–base and electrolyte balances, and fluid status^5^. The clearance of urea remains the most strongly recommended measurement of dialysis adequacy in clinical practice^5^, with measurements including Kt/V and URR (urea reduction ratio). Kt/V [K: dialyzer clearance (mL/min); t: dialysis time (min); V: urea distribution volume (mL)] was developed by Frank Gotch and John Sargent^6^ and further equilibrated by Daugirdas in the 1990s^7^;it remains the primary indicator of dialysis adequacy^5,8^. A landmark randomized controlled trial in 2002, the HEMO study, found that single-pool Kt/V (spKt/V) > 1.2 is associated with lower mortality in HD patients^9^. The KDOQI (Kidney Disease Outcomes Quality Initiative) guidelines recommend a target spKt/V of 1.4 per HD session for patients treated three times weekly, with a minimum delivered spKt/V of 1.2^5^. URR is calculated using a relatively simple equation developed by Lowrie and Lew in 1991^10^. The recommended dosage of URR ranges from > 65% to > 75% according to different studies^11,12^. Taiwan Society of Nephrology uses URR in clinical practice and suggests URR > 65% as a minimum requirement.

Due to reasons include the narrower range of doses achieved during HD for URR compare to Kt/V, the variation of curvilinear relationship between the two parameters (because Kt/V considers urea distribution volume and UF)^13^ and URR decreases substantially during continuous renal replacement therapy^8^. Kt/V has long been preferred to URR as the standard for prescribing HD dosage and is recommended by the KDOQI^5,8^. However, Kt/V also has its potential disadvantages which may overcome its benefits and has been discussed in recent decades.

Kt/V, which considers volume of urea distribution in the body, is thought to be more accurate than URR. However, V and Kt are both considered survival-associated factors, which may cause an offset effect^14,15^. For example, for patients with lower mass, high Kt/V occurs more easily due to lower V, which causes overestimation of dialysis dosage^16,17^. Although studies have attempted to establish an association between Kt/V and URR, measurements cannot be converted accurately due to ultrafiltration (UF) level and dialysis time ^8,18^. The prognostic value of Kt/V is theoretically greater than that of URR, but head-to-head comparison has not been made.

The prognostic value of Kt/V is modified in some subgroups. Patients with low V may have high Kt/v. Studies have shown that body weight modifies the association between Kt/v and all-cause mortality^19^, but the results of some studies are inconsistent^12^. Some studies also showed gender, body size and physical activity can all influent the dialysis dose and mortality^20,21^. Others have found that, female sex, but not low body weight, modified this effect^22^. Nutritional status may also modify this association^23^, and cardiovascular disease may modify the association between Kt/v and cardiac outcomes^24^. We thus investigated whether low V modified the association between URR and all-cause mortality and compared the results of subgroup analysis between URR and Kt/v.

We thus examined and compared the prognostic value of Kt/V with that of URR for all-cause mortality and compared the factors modifying this effect in incident HD patients in southern Taiwan. In subgroup analysis, we stratified the patients according to sex, age, body weight, comorbidities, and laboratory data. We used the averaged dialysis dose between months 4 and 9 in incident HD patients to prevent bias.

## Results

### Clinical characteristics of incident HD

Table 1 shows baseline characteristics of the cohort. In 2651 incident HD patients, the mean age was 59.1 ± 14.2 years. Half of the population was female (50.4%), and the most common comorbidities were hypertension (70%), DM (48.2%), and CHF (32.5%). Patients had a mean dialysis dose of 72% ± 7% URR and 1.55 ± 0.29 Kt/V, with a mean UF of 2.2 ± 1.0 L per HD session. The mean HD session length was 238.9 ± 6.6 min. The mortality rate was 33.1%. We further stratified the cohort by URR and Kt/V levels in Table 1.

**Table 1:** Baseline characteristics of patients stratified by URR and Kt/V levels.

Number (%)	URR						Kt/V					
	All	< 65%	65–70%	70–75%	> 75%	P value	All	< 1.2	1.2–1.4	1.4–1.7	> 1.7	P value
	2615	408 (15.6)	580 (22.2)	812 (31.1)	815 (31.2)		2615	267 (10.2%)	543 (20.8%)	1023(39.1%)	782 (29.9%)	
Demographics												
Age at dialysis	59.1 (14.2)	55.2 (13.8)	58.0 (14.1)	59.1 (14.0)	61.6 (14.0)	< 0.001	59.1 (14.2)	56.0 (14.0)	57.81 (14.2)	59.08 (14.0)	60.93 (14.2)	< 0.001
Entry year	1539 (58.9%)	231 (56.6%)	351 (60.5%)	470 (57.9%)	487 (59.8%)	0.552	1539 (58.9%)	152 (56.9%)	327 (60.2%)	606 (59.2%)	454 (58.1%)	0.778
Female (%)	1317 (50.4)	90 (22.1)	162 (27.9)	402 (49.5)	663 (81.3)	< 0.001	1317 (50.4)	63 (23.6)	129 (23.8)	487 (47.6)	638 (81.6)	< 0.001
Comorbidities												
Hepatitis	361 (13.8%)	64 (15.7%)	91 (15.7%)	107 (13.2%)	99 (12.1%)	0.164	361 (13.8%)	44 (16.5%)	91 (16.8%)	132 (12.9%)	94 (12.0%)	0.038
CHF	850 (32.5%)	160 (39.2%)	209 (36.0%)	237 (29.2%)	244 (29.9%)	< 0.001	850 (32.5%)	107 (40.1%)	195 (35.9%)	305 (29.8%)	243 (31.1%)	0.003
Stroke	194 (7.4%)	28 (6.9%)	46 (7.9%)	56 (6.9%)	64 (7.9%)	0.813	194 (7.4%)	18 (6.7%)	41 (7.6%)	80 (7.8%)	55 (7.0%)	0.896
Cancer	161 (6.2%)	22 (5.4%)	38 (6.6%)	34 (4.2%)	67 (8.2%)	0.007	161 (6.2%)	19 (7.1%)	36 (6.6%)	48 (4.7%)	58 (7.4%)	0.086
Diabetes mellitus	1261 (48.2%)	245 (60.0%)	285 (49.1%)	411 (50.6%)	320 (39.3%)	< 0.001	1261 (48.2%)	150 (56.2%)	288 (53.0%)	515 (50.3%)	308 (39.4%)	< 0.001
Hypertension	1831 (70.0%)	290 (71.1%)	430 (74.1%)	565 (69.6%)	546 (67.0%)	0.036	1831 (70.0%)	185 (69.3%)	394 (72.6%)	726 (71.0%)	526 (67.3%)	0.171
Hemodialysis parameters												
BW before HD (kg)	58.9 (12.0)	68.7 (14.3)	63.6 (10.4)	57.9 (9.9)	51.5 (8.2)	< 0.001	58.9 (12.0)	69.0 (15.3)	65.2 (11.7)	58.6 (9.5)	51.3 (8.3)	< 0.001
BW after HD (kg)	56.6 (11.6)	66.1 (13.9)	61.2 (10.1)	55.7 (9.6)	49.6 (8.1)	< 0.001	56.6 (11.6)	66.5 (14.7)	62.9 (11.3)	56.4 (9.2)	49.2 (8.1)	< 0.001
UF amount (kg)	2.24 (0.96)	2.62 (1.08)	2.37 (0.95)	2.24 (0.96)	1.96 (0.80)	< 0.001	2.24 (0.96)	2.49 (1.16)	2.32 (0.92)	2.23 (0.88)	2.13 (1.00)	< 0.001
UF/BW ratio * 100	3.82 (1.50)	3.83 (1.50)	3.74 (1.42)	3.88 (1.58)	3.83 (1.48)	0.384	3.82 (1.50)	3.60 (1.56)	3.55 (1.32)	3.79 (1.38)	4.13 (1.69)	< 0.001
URR	0.72 (0.07)	0.60 (0.07)	0.68 (0.01)	0.73 (0.01)	0.79 (0.03)	–	0.72 (0.07)	0.59 (0.09)	0.67 (0.03)	0.72 (0.03)	0.78 (0.03)	–
Kt/V (Daugirdas)	1.55 (0.29)	1.16 (0.17)	1.38 (0.10)	1.57 (0.14)	1.86 (0.19)	–	1.55 (0.29)	1.07 (0.14)	1.31 (0.06)	1.55 (0.08)	1.89 (0.17)	–
nPCR	1.15 (0.28)	1.06 (0.30)	1.10 (0.26)	1.16 (0.28)	1.23 (0.28)	< 0.001	1.15 (0.28)	1.04 (0.30)	1.10 (0.27)	1.16 (0.28)	1.22 (0.28)	< 0.001
Type of access												
AVF	1794 (68.6%)	280 (68.6%)	420 (72.4%)	561 (69.1%)	533 (65.4%)	< 0.001	1794 (68.6%)	177 (66.3%)	401 (73.8%)	704 (68.8%)	512 (65.5%)	< 0.001
AVG	482 (18.4%)	59 (14.5%)	78 (13.4%)	165 (20.3%)	180 (22.1%)	–	482 (18.4%)	39 (14.6%)	71 (13.1%)	201 (19.6%)	171 (21.9%)	–
Surface area of dialyzer (m^2^)												
1.2–1.6	600(32.6%)	67 (22.9%)	100(24.3%)	174(30.1%)	259(46.5%)	< 0.001	600 (32.6%)	45 (23.1%)	95 (23.5%)	208 (28.9%)	252 (48.4%)	< 0.001
1.7–1.9	944(51.3%)	130(44.4%)	221(53.6%)	337(58.2%)	256(46.0%)	-	944 (51.3%)	90 (46.2%)	205 (50.6%)	416 (57.8%)	233 (44.7%)	–
2.0–2.2	297(16.1%)	96 (32.8%)	91 (22.1%)	68 (11.7%)	42 (7.5%)	-	297 (16.1%)	60 (30.8%)	105 (25.9%)	96 (13.3%)	36 (6.9%)	–
Dialyzer classification												
Low-flux dialyzers	33 (1.3%)	9 (2.2%)	8 (1.4%)	10 (1.2%)	6 (0.7%)	–	33 (1.3%)	7 (2.6%)	9 (1.7%)	12 (1.2%)	5 (0.6%)	–
High-flux dialyzers	2582 (98.7%)	399 (97.8%)	572 (98.6%)	802 (98.8%)	809 (99.3%)	–	2582 (98.7%)	260 (97.4%)	534 (98.3%)	1011 (98.8%)	777 (99.4%)	–
Laboratory data												
WBC (× 1000/ul)	7.00 (2.27)	7.22 (2.40)	7.25 (2.47)	7.07 (2.23)	6.66 (2.06)	< 0.001	7.00 (2.27)	7.12 (2.44)	7.33 (2.52)	7.05 (2.24)	6.68 (2.03)	< 0.001
Hb (g/dl)	9.85 (1.22)	9.86 (1.31)	9.92 (1.27)	9.83 (1.16)	9.82 (1.21)	0.469	9.85 (1.22)	9.83 (1.34)	9.95 (1.25)	9.86 (1.19)	9.79 (1.19)	0.144
Albumin (gm/dl)	3.74 (0.41)	3.68 (0.44)	3.76 (0.43)	3.75 (0.40)	3.74 (0.37)	0.011	3.74 (0.41)	3.67 (0.45)	3.74 (0.45)	3.76 (0.39)	3.74 (0.37)	0.013
Total chol. (mg/dl)	172 (116)	184 (126)	176 (113)	179 (124)	156 (103)	< 0.001	172 (116)	182 (124)	180 (116)	175 (118)	159 (109)	0.002
Glucose[AC](mg/dl)	136 (60)	146 (63)	138 (59)	138 (63)	127 (57)	< 0.001	136 (60)	145 (64)	139 (58)	137 (61)	130.1 (59)	< 0.001
BUN (mg/dl)	70.3 (18.1)	72.7 (20.5)	70.4 (18.1)	69.7(18.1)	69.5(16.7)	0.017	70.3 (18.1)	73.0 (20.8)	71.1 (19.2)	69.7 (17.6)	69.4 (16.8)	0.022
Creatinine (mg/dl)	9.25 (2.84)	10.03(3.34)	9.82 (3.09)	9.22 (2.70)	8.48 (2.26)	< 0.001	9.25 (2.84)	9.93 (3.51)	9.91 (3.14)	9.29 (2.70)	8.51 (2.32)	< 0.001
CTR100	50.31 (6.50)	50.04 6.51)	49.54(6.48)	50.34(6.48)	50.98(6.48)	< 0.001	50.31 (6.50)	50.26 (6.26)	49.76 (6.58)	50.24 (6.52)	50.81 (6.48)	0.032
Iron saturation	32.3 (20.5)	31.5 (30.4)	31.6 (19.9)	32.6 (19.0)	33.0 (15.7)	0.501	32.3 (20.5)	31.3 (20.1)	32.2 (29.2)	32.3 (18.4)	32.9 (15.4)	0.746
P (mg/dl)	4.97 (1.24)	5.26 (1.41)	5.01 (1.25)	4.97 (1.18)	4.79 (1.17)	< 0.001	4.97 (1.24)	5.22 (1.45)	5.05 (1.34)	4.97 (1.17)	4.83 (1.17)	< 0.001
Clinical outcome												
Follow up days	2261 (1301)	2131 (1354)	2227 (1308)	2317 (1281)	2295 (1286)	0.087	2261 (1301)	2088 (1380)	2210 (1295)	2304 (1292)	2299 (1287)	0.06
Mortality (10y)	866 (33.1%)	160 (39.2%)	190 (32.8%)	252 (31.0%)	264 (32.4%)	0.034	866 (33.1%)	110 (41.2%)	183 (33.7%)	326 (31.9%)	247 (31.6%)	< 0.05

Data are presented as mean (standard deviation), median (interquartile range), or count (percentage %).

*CHF* congestive heart failure, *BW* body weight, *HD* hemodialysis, *UF* ultrafiltration, *URR* urea reduction ratio, *nPCR* normalized protein catabolism rate,*AVF* arteriovenous fistula, *AVG* arteriovenous graft, *WBC* white blood cell, *Hb* hemoglobin, *Total chol*. total cholesterol, *CTR* cardiothoracic ratio.

### Clinical characteristics of incident HD patients stratified by URR and Kt/V levels

After stratifying the cohort in Table 1 by URR (< 65%, 65–70%, 70–75%, > 75%) and Kt/V (< 1.2, 1.2–1.4, 1.4–1.7,  > 1.7) levels, we found that more than half of the pa-tients had higher dialysis dose: 62.3% of patients had URR > 70%, and 69% of patients had Kt/V > 1.4. High-flux dialyzers were used in most of the patients during hemodialysis (98.7%), while low-flux dialyzers were more in patients with low URR or low Kt/V, but in very low percentage (1.3%). We observed that higher URR or Kt/V levels were associated with fe-male sex, higher age, absence of diabetes, and lower BW. Higher URR and Kt/V levels were both associated with higher nutritional markers (nPCR and total cholesterol) but lower WBC count and serum creatinine.

### Factors associated with URR and Kt/V

Table 2 shows the multivariate linear regression for URR and Kt/V. URR and Kt/V both correlated positively with female sex, nPCR, and albumin. Conversely, URR and Kt/V correlated negatively with CHF and BW.

**Table 2:** Multivariate linear regression for URR and Kt/V.

	URR per SD		P value	Kt/V per SD		P value
	β coefficient	95% CI		β coefficient	95% CI	
Constant	− 1.017			− 0.457		
Gender (female)	0.544	0.473 to 0.614	< 0.001	0.588	0.519 to 0.657	< 0.001
Entry year	0.008	− 0.062 to 0.077	0.831	0.029	− 0.039 to 0.096	0.402
DM	− 0.103	− 0.176 to -0.029	0.006	− 0.061	− 0.133 to 0.010	0.094
Hypertension	0.021	− 0.050 to 0.092	0.564	0.011	− 0.058 to 0.079	0.763
CHF	− 0.116	− 0.185 to − 0.046	0.001	− 0.104	− 0.172 to − 0.036	0.003
Stroke	0.091	− 0.028 to 0.211	0.135	0.052	− 0.065 to 0.169	0.382
Cancer	0.053	− 0.077 to 0.182	0.426	0.054	− 0.072 to 0.180	0.399
Hb (g/dl)	0.013	− 0.016 to 0.041	0.382	0.014	− 0.013 to 0.042	0.301
BW after HD(kg)	− 0.032	− 0.035 to − 0.028	< 0.001	− 0.037	− 0.040 to − 0.034	< 0.001
nPCR	0.638	0.521 to 0.756	< 0.001	0.541	0.427 to 0.656	< 0.001
CTR 100	− 0.000	− 0.005 to 0.005	0.959	− 0.002	− 0.007 to 0.003	0.418
Albumin (gm/dl)	0.446	0.351 to 0.540	< 0.001	0.394	0.302 to 0.486	< 0.001
W.B.C. (× 1000/ul)	− 0.008	− 0.023 to 0.006	0.266	− 0.010	− 0.024 to 0.004	0.162
Cholesterol log	− 0.248	− 0.571 to 0.076	0.133	− 0.202	− 0.516 to 0.112	0.208
Glucose[AC](mg/dl)	− 0.000	− 0.001 to 0.000	0.217	− 0.000	− 0.001 to 0.000	0.661
P (mg/dl)	− 0.050	− 0.079 to − 0.021	0.001	− 0.025	− 0.053 to 0.004	0.087
Iron saturation	0.001	− 0.001 to 0.002	0.254	0.000	− 0.001 to 0.002	0.671

Multivariate linear regression model showing the Beta Coefficient and the p value of the Association with the variable.

*URR* urea reduction ratio, *CI* confidence interval, *SD* standard deviation, *DM* diabetes mellitus, *CHF* congestive heart failure, *Hb* hemoglobin, *BW* body weight, *HD* hemodialysis, *nPCR* normalized protein catabolism rate, *CTR* cardiothoracic ratio, *WBC* White blood cell.

### URR, Kt/V, and all-cause mortality

In total, 866 deaths (33.1%) occurred during a median 5.02-year follow-up period. Based on the literature^25,26^, we performed Cox survival analysis using age at dialysis, sex, entry year, DM, hypertension, hepatitis, postdialysis BW, nPCR, UF/BW ratio, creatinine, hemoglobin, WBC, albumin, AC, log-transformed cholesterol, and phosphorus (Table 3). After stratifying patients by URR and Kt/V levels, 160 (39.2%), 190 (32.8%), 252 (31.0%), and 264 (32.4%) deaths occurred in the URR < 65%, 65–70%, 70–75%, and > 75% groups, respectively, and 110 (41.2%), 183 (33.7%), 326 (31.9%), and 247 (31.6%) deaths occurred in the Kt/V < 1.2, 1.2–1.4, 1.4–1.7, and > 1.7 groups, respectively. The lowest mortality rates among patients were of those who received URR of 70–75% and Kt/V > 1.7. Mortality rates were significantly higher for all cohort populations withURR < 70% vs > 70% (35.4% vs 31.7%)and Kt/V < 1.4 vs > 1.4(36.1% vs 31.7%; Table 1). In all study populations after adjustment, both higher URR (70–75%, > 75%)and Kt/V (1.4–1.7, > 1.7) groups had lower risk of all-cause mortality with adjusted hazard ratios (HRs) of 0.693 (95% confidence interval CI 0.578–0.829, *P* < 0.001) and 0.640 (95% CI 0.519–0.788, *P* < 0.001) in the URR group and 0.656 (95% CI 0.540–0.799, *P* < 0.001) and 0.623 (95% CI 0.498–0.779, *P* < 0.001) in the Kt/V group, compared with the URR < 65% and Kt/V < 1.2 groups. Although no matter higher Kt/V or URR groups both associated with lower HR, the effect of reduction of HR attenuating as in higher Kt/V or URR group. Moreover, each1SD increase in URR and Kt/V was associated with HRs of 0.896 (95% CI 0.844–0.952, *P* < 0.001) and 0.885(95% CI 0.824–0.952, *P* < 0.001), respectively (Table 3).

**Table 3:** Hazard ratios for mortality according to URR and Kt/V groups.

	Per 1SD increase of URR	URR				P for trend
		< 65%	65–70%	70–75%	> 75%	
Unadjusted	0.915 (0.868–0.964)**	1 (reference)	0.849 (0.711–1.014)	0.809 (0.686–0.956)*	0.774 (0.655–0.916)*	0.022
Fully-adjusted	0.896 (0.844–0.952)**	1 (reference)	0.748 (0.623–0.898)*	0.693 (0.578–0.829)**	0.640 (0.519–0.788)**	< 0.001

	Per 1SD increase of Kt/V	Kt/V				P for trend
		< 1.2	1.2–1.4	1.4–1.7	> 1.7	
Unadjusted	0.914 (0.863–0.967)*	1 (reference)	0.821 (0.672–1.005)	0.771 (0.641–0.927)*	0.732 (0.604–0.887)*	0.012
Fully-adjusted	0.885 (0.824–0.952)**	1 (reference)	0.711 (0.580–0.873)*	0.656 (0.540–0.799)**	0.623 (0.498–0.779)**	< 0.001

95% confidence interval (CI).

Adjusted model: adjusted for age at dialysis, gender, entry year, DM, hypertension, hepatitis, BW after dialysis, nPCR, UF/BW ratio, creatinine, hemoglobin, WBC, albumin, glucose, cholesterol log, phosphorus.

*URR* urea reduction ratio, *SD* standard deviation.

*P < 0.05 compared with reference URR or Kt/V category.

**P < 0.001 compared with reference URR or Kt/V category.

### Subgroup analysis by URR and Kt/V

**Figure 1:**
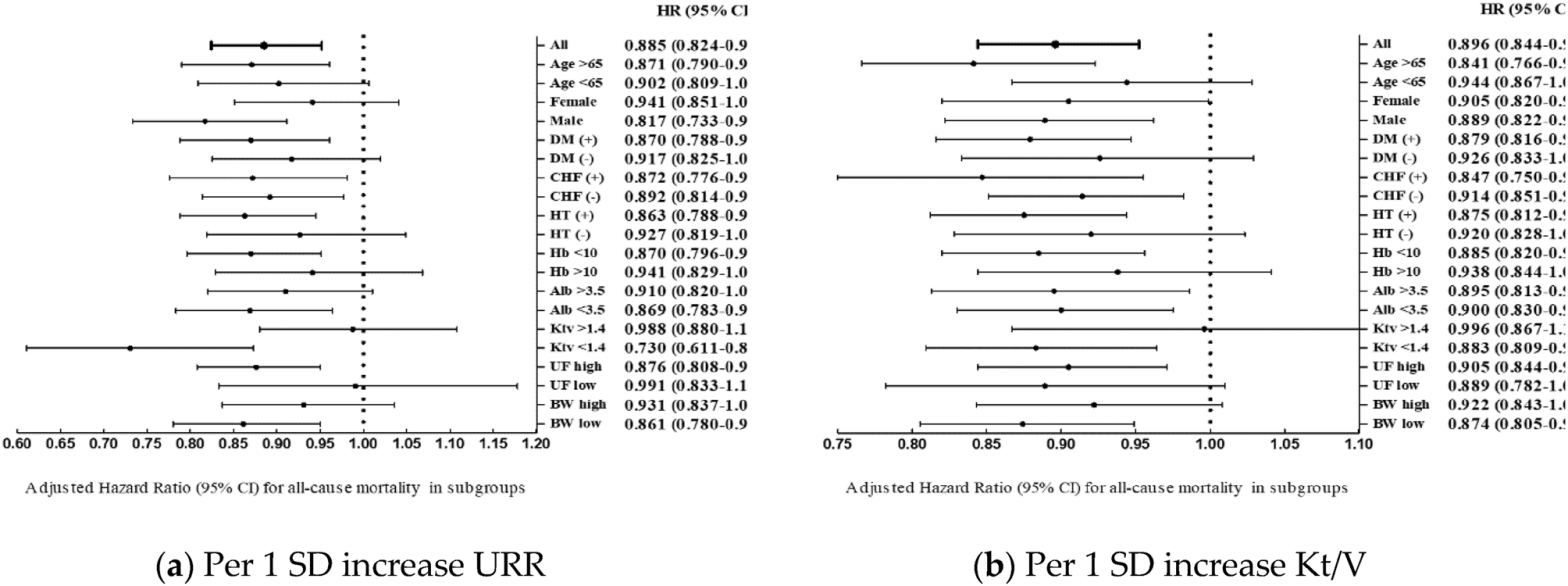
(**a**) Adjusted HR (95% Cl) for all-cause mortality in subgroups according to URR; (**b**) adjusted HR (95% CI) for all-cause mortality in subgroups according to Kt/V. *URR* urea reduction ratio, *SD* standard deviation, *CI* confidence in-terval, *HR* hazard ratio, *DM* diabetes mellitus, *CHF* congestive heart failure, *HT* hypertension, *Hb* hemoglobin, *Alb* albumin, *UF* ultrafiltration, *BW* body weight.

Figure 1 shows adjusted HRs (95% CI) for all-cause mortality in subgroups ac-cording to URR (Fig. 1a) and Kt/V (Fig. 1b). HRs for each1SD increase of URR and Kt/V by subgroups, including those of age, sex, DM, CHF, hypertension, hemoglobin, albumin, and BW, were all analyzed and compared. In both URR and Kt/V groups, each1SD increase in URR and Kt/V was associated with mortality with a low dialysis dose but not with a high dialysis dose (P < 0.05). A 1SD increase in Kt/V was not associ-ated with HRs in women(0.941, 95% CI 0.51–1.041) but was in men (0.817, 95% CI 0.733–0.911; P < 0.05).

## Disccussion

We examined the prognostic value of URR versus Kt/V for all-cause mortality and compared the factors modifying this effect in 2615 incident HD patients in southern Taiwan. We determined that higher dialysis dose (Kt/V > 1.2, single-pool Daugirdas formula or URR > 65%) were both associated with lower all-cause mortality compared with lower dialysis dose (Kt/V < 1.2, single-pool Daugirdas formula or URR < 65%). Moreover, in subgroup analysis, we found that higher dialysis dose was significantly associated with mortality in those with Kt/V < 1.4 but not in those with Kt/V ≥ 1.4. Kt/V was not associated with mortality in women.

Dialysis inadequacy is one of the main causes of mortality in HD patients^1,2,27^. The clearance of small molecules such as urea, Kt/V, and URR is critical for dialysis adequacy evaluation. Optimal dialysis and the relation between dialysis adequacy and survival have been heavily researched, although no randomized studies investigating minimum dialysis dosage have been undertaken. Several retrospective studies have demonstrated that Kt/V > 1.2 is associated with better survival^28,29^. Studies have also suggested targeting Kt/V > 1.4 to achieve a minimum of Kt/V > 1.2 due to barriers to adequate delivery, such as lower blood flow, shorter time, recirculation, and use of a catheter for vascular access^30,31^. The KDOQI Clinical Practice Guideline for Hemodialysis Adequacy^8^ suggests a minimum URR dose of > 65% and a target dose of > 70% for patients receiving HD three times per week with treatment times less than 5 hours^8^. Barriers to URR and Kt/V correlation have been indicated, including higher UF, which may cause increased Kt/V, and long dialysis time, which may cause decreased URR^8,18^. Another study found that after stratifying patients into three BMI (body mass index) groups (low, medium, and high), the relative risk (RR) decreased when URR increased. Furthermore, patients treated with URR > 75% had a substantially lower RR than patients treated with URR 70–75% (*P* < 0.005 for medium and low BMI groups)^12^. Although patients benefit from higher Kt/V or URR, Chertowet al. demonstrated that patients with extremely high URR (> 75%) or single-pooled Kt/V (> 1.6) may be more severely malnourished, which may increase mortality and limit the utility of URR or Kt/V^32^. In our study, we found that URR > 70% or Kt/V > 1.4 was associated with better survival. In the subgroup analysis, we found that higher dialysis dose was significantly associated with mortality in those with Kt/V < 1.4 but not in those with Kt/V ≥ 1.4 which is compatible with the attenuation of HR we found in higher Kt/V or URR group in Table 3. However careful evaluation of the nutritional status of patients is also crucial.

Kt/V has long been preferred to URR as the standard for prescribing HD dosage and is recommended by the KDOQI. The reasons include the narrow range of doses achieved during HD, the curvilinear relationship between the two parameters (because Kt/V considers urea distribution volume and UF), that URR decreases substantially during continuous renal replacement therapy or daily dialysis whereas the given Kt/V remains the same, and the inability to account for the effect of residue kidney function with URR^8,13^. However, Kt/V has some disadvantages compared with URR. First, V and Kt are both considered survival-associated factors, which may cause an offset effect^14,15^. For example, in patients with lower mass, high Kt/V is easier to achieve due to low V, which causes overestimation of dialysis dose^16^, even when compared with using Kt only^17^. Second, models of Kt/V assume stable outpatient status with end-stage renal disease, which does not apply in situations such as those of critically ill patients with acute kidney injury whose V may exceed total body water and may vary significantly^15^. Therefore, when outcomes, including death, are correlated with URR and Kt/V, no difference in degree of correlation is detectable^8^.

In subgroup analysis, we found that factors such as age, BW, and other comorbidities did not modify the association between either Kt/v or URR and all-cause mortality, except for sex in the Kt/V group. We found that increased Kt/V was not associated with mortality in women. To explain this sex difference, studies have indicated that underdialysis occurs more in women. In recent studies, the various minimum target dialysis does of Kt/V was found in sedentary patients by gender and body size after using alternative parameters such as Kt/TEE(Total energy expenditure) and Kt/BSA(Body surface area), woman and smaller man were found generally underdialysis by using Kt/V^20,21^. In another recent study in Gulf Cooperation Council (GCC) population, they found low Kt/V was strongly related to higher mortality in women, but not in men^33^. Perez-Garcia et al. demonstrated that patients with a higher Kt/V, which occurs more easily in women, had lower survival rates than others. Higher Kt/V values are due to a lower V and poorer nutritional status^34^. Another study determined that the increased mortality with extremely high URR (> 75%) or single-pooled Kt/V (> 1.6) values (manifested in a lower V) due to malnutrition outweighed the benefits of greater urea clearance^32^. Moreover, studies also found that female HD patients tended to have more severe malnutrition^35^ and lower albumin levels^36^,outweighing the benefits of relatively high Kt/V. Due to this malnutrition factor, it is difficult to evaluate the actual benefits of increased dialysis dosage in women by using Kt/V. Our study found that sex modified the association between either Kt/V or URR and all-cause mortality, but BW did not. However, the conversion of BW to V was based on the Watson formula, which was designed in relation to a healthy Western population and might not reflect actual V in our study population^37,38^. In a later analysis in the HEMO study, increasing dialysis dose (double-pool Kt/V 1.53 vs double-pool Kt/V 1.16) in a subgroup of women reduced mortality by 19%; it did not cause a significant difference in men. This result persisted after adjustment for the interaction of dosage with body water volume or with other mass parameters, including weight and body mass index, which indicates that factors other than body size may have contributed to this result^22^. Another study found a similar sex difference in mortality benefit for women on HD in Japan with spKt/V levels ≥ 1.6^39^. Although the previous studies were all favorable of underdialysis of woman in Kt/V, which is opposite to our result that no obvious prognosis value for woman with higher Kt/V, but in man. However, these studies all undeniably point out that sex difference did exist when using Kt/V. The mechanism of the sex difference modifying the association between Kt/V and all-cause mortality in our study remains unclear and maybe relate to race or specific population in our study. However, an optimal indicator for mortality should not generally be affected by age, sex, or comorbidities. Therefore, URR may have prognostic value for mortality equal to or greater than Kt/V, as shown in this study.

This study enrolled incident HD patients and measured average dialysis dose between months 4 and 9 of HD. This may have prevented survival bias and ensured stable measurement of dialysis dose. This study has limitations. First, it was an observational study, and a causal relationship between Kt/V or URR and clinical outcomes could not be established. Second, residual urine was not measured. However, as in our previous report, our patients with chronic kidney disease started dialysis late, at a mean eGFR of 5 mL/mL/1.73m^2^ with considerably low urine output^40^. Third, some other data were absent, such as blood pressure, medication, and BMI. Fourth, Kt, BSA, TEE records were absent and thus could not be compared with Kt/V.

## Conclusions

Our study demonstrated that in a head-to-head comparison between URR and Kt/V in 2651 incident HD patients, URR has as much prognostic value for mortality as Kt/V in this study population (Asian or Taiwanese population). Moreover, compared with URR, Kt/V appeared to have less prognostic value in the female subgroup. Finally, higher URR (URR > 70%) and Kt/V (Kt/V > 1.4) were both associated with lower all-cause mortality in incident HD patients. Larger studies to compare the prognostic value of URR and Kt/V for mortality and more application of URR in the future studies are necessary. In addition, the prognostic value of Kt/V in women may warrant further investigation.

## Materials and Methods

### Participants and design

The Kaohsiung Hemodialysis Study was designed as a prospective cohort study to investigate the quality of patient care on the basis of the HD, Operation, Plan, Executive management system developed by the Taiwan Society of Nephrology. Between 1 January 1997 and 31 December 2009, 2748 consecutive incident HD patients who had stable thrice-weekly dialysis for more than 90 days and were aged more than 18 years from three affiliated hospitals and nine associated HD clinics of Kaohsiung Medical University in southern Taiwan were included and followed up until 31 May 2013. Of these, 94 patients discontinued follow-up within 6 months, and 39 patients had more than 10% data missing. The final study population consisted of 2615 incident HD patients. The study was conducted according to the guidelines of the Declaration of Helsinki, and ap-proved by the Institutional Review Board of Kaohsiung Medical University Hospital (KMUH-IRB-990198). We have confirmed that informed consent was obtained from all subjects and/or their legal guardian(s).

Dialysis initiation was carried out according to the regulations of the National Health Insurance Administration of Taiwan, which specify required laboratory data, nutritional status, uremic status, and estimated glomerular filtration rate (eGFR). Dialysis initiation was late with a mean eGFR of 4.9 mL/min/1.73 m^2^ and a mean residual urine of 560 mL. In total, 178 patients (6.1%) were receiving incident peritoneal dialysis at the same period. The National Health Insurance Administration provides total coverage of HD treatment and erythropoiesis-stimulating agent treatment with a fixed payment. The attending physicians rotated between the dialysis centers, and dialysis machines were also rotated; artificial kidneys and water management were similar.

In addition, we do not lower the surface area dialyzer if target URR(70%) or Kt/V (> 1.4) as suggested by KDIGO achieved. However, we will increase the surface area dialyzer if the minimal requirement of URR(> 65%) or Kt/V (> 1.2) were not achieved according to the guideline of Taiwan Society of Nephrology.

### Measurements

Baseline variables included demographic features (age, sex, and entry year), medical history (diabetes mellitus [DM], congestive heart failure [CHF], hypertension, stroke, cancer, and hepatitis). We collected and averaged examination findings (pre- and postdialysis body weight [BW]), laboratory data (blood creatinine, postdialysis blood urea nitrogen[BUN], albumin (Bromocresolgreen(BCG) albumin assay via Roche cobas® 6000 analyzer), white blood cell count [WBC], hemoglobin, total cholesterol, cardiothoracic ratio [CTR], iron saturation, and glucose[AC]) and HD parameters (UF/BW ratio, vascular access, Kt/V[Daugirdas], URR, and normalized protein catabolic rate [nPCR])between months4 and 9. Demographic features were obtained from baseline records, and medical history was obtained by reviewing doctors’ charts. DM and hypertension were defined by clinical diagnosis. Laboratory data were collected monthly, and the mean data from6 months after stable dialysis were used in analysis. Dialysis adequacy wasmeasured using the single-pool Daugirdas formula Kt/V =  − 1n((postBUN/preBUN) − 0.008 × t) + [(4 − 3.5 × (postBUN/preBUN)) × UF/BW] and URR = (preBUN − postBUN)/preBUN(BUN: mg/dL). Postdialysis BUN was drawn according to Kidney Disease Outcomes Quality Initiative (KDOQI) guidelines as follows^8^: (1) The ultrafiltration rate was set to zero, (2) the blood pump was slowed to 100 mL/min for 10–20 s, (3) the pump was then stopped and (4) a sample was drawn either from an arterial blood line sampling port or from tubing attached to the arterial needle^41,42^.

### Outcomes

Patients were followed up from month 4 to the end of month 20 of HD or death. All-cause mortality was ascertained by review of death certificates using charts or the National Death Index.

### Statistical analysis

The PA Baseline characteristics were expressed as percentages for categorical data, mean ± standard deviation (SD) for continuous variables with approximately normal distribution, and median and interquartile ranges for continuous variables with skewed distribution. The Markov chain Monte Carlo method was used to mitigate the effects of missing covariates (less than 5% missing values for seven covariates). Multivariate linear regression was used to evaluate the relationship between URR, Kt/V, and the significant factors listed in Table 1. Cox proportional hazards analysis was used to evaluate the relationship between URR, Kt/V, and mortality. Analysis was performed initially without adjustment but was subsequently adjusted for several groups of covariates stratified by our study. Covariates with *P* < 0.05 in univariate analysis were included in these models, and skewed distribution continuous variables were log transformed to attain normal distribution. These were age at dialysis initiation, sex, entry year, DM, hypertension, hepatitis, CHF, postdialysis BW, nPCR, UF:BW ratio, creatinine, hemoglobin, WBC, albumin, AC, log-transformed cholesterol, and phosphorus. Prespecified subgroup analyses were also performed in patients stratified by sex, age (≥ 65 years), DM, CHF, hepatitis, hypertension, anemia (hemoglobin < 10 g/dL), albumin (< 3.5 g/dL), UF (mean), and BW (mean). Interactions between subgroups were tested. A *P* value < 0.05 indicated significance. Statistical analysis was performed using R 4.1.3 software (R Foundation for Statistical Computing, Vienna, Austria) and SPSS version 20.0 (SPSS Inc., Chicago, IL).

### Ethics declarations

The study was conducted according to the guidelines of the Declaration of Helsinki, and ap-proved by the Institutional Review Board of Kaohsiung Medical University Hospital (KMUH-IRB-990198).

## Data Availability

Study data are available from the corresponding author (C.-C.H.) upon request.
